# Impact of multiplex polymerase chain reaction testing in patients with bacteremia

**DOI:** 10.1128/spectrum.01980-25

**Published:** 2025-09-24

**Authors:** Daisuke Kitagawa, Taito Kitano, Takehito Kasamatsu, Naoyuki Shiraishi, Mai Yasuda, Mai Okada, Soma Suzuki, Madoka Sekine, Ryo Yamanishi, Ayu Mukai, Ritsuki Uejima, Yuki Suzuki, Akiyo Nakano, Ryuichi Nakano, Hisakazu Yano, Fumihiko Nakamura, Koichi Maeda

**Affiliations:** 1 Department of Laboratory Medicine, Nara Prefecture General Medical Center 36927, Nara, Japan; 2 Department of Microbiology and Infectious Diseases, Nara Medical University 12967, Kashihara, Japan; 3 Department of Pediatrics, Nara Prefecture General Medical Center 36927, Nara, Japan; 4 Department of Infectious Diseases, Nara Prefecture General Medical Center 36927, Nara, Japan; 5 Department of Pharmacy, Nara Prefecture General Medical Center 36927, Nara, Japan; Ann & Robert H. Lurie Children's Hospital of Chicago, Chicago, Illinois, USA

**Keywords:** bacteremia, FilmArray blood culture identification 2 (BCID2), days of antimicrobial spectrum coverage (DASC), antimicrobial days of therapy (DOT), active antimicrobial stewardship programs (ASP)

## Abstract

Rapid identification of pathogens in bacteremia is critical for optimizing antimicrobial therapy. The FilmArray blood culture identification 2 (BCID2) panel enables multiplex PCR-based detection of pathogens and resistance genes. This study aimed to evaluate the clinical impact of BCID2 implementation in hospitalized patients with bacteremia. To this end, we conducted a retrospective study at a single Japanese center (May 2018–December 2024). The outcomes included length of stay, in-hospital mortality, antimicrobial days of therapy (DOT), and days of antimicrobial spectrum coverage (DASC) score. The pre-BCID2 and BCID2 implementation periods were compared. Among the 2,872 patients with positive blood cultures, BCID2 implementation was associated with a significant reduction in mortality (adjusted odds ratio [aOR] 0.58, 95% CI: 0.37–0.92, *P* = 0.021), specifically among those with bacteremia during weekday daytime hours when testing was available (aOR: 0.50, 95% CI: 0.28–0.89, *P* = 0.018). Age, sex, and intensive care unit (ICU)/high-care unit (HCU) admission were significant predictors of outcomes across all analyses. Implementing the BCID2 panel during weekday daytime hours within an active antimicrobial stewardship program (ASP) could significantly reduce mortality among patients with bacteremia when testing was available; however, it showed no benefits in the overall population. The findings of this study emphasize the importance of continuous availability and effective integration with ASP support to optimize clinical benefits.

**IMPORTANCE:**

Rapid identification of the bacteria causing bloodstream infections is essential for timely and effective treatment. Traditional laboratory methods are often time-consuming, thus delaying therapy. Therefore, this study examined the application of the FilmArray blood culture identification panel, a rapid molecular test that can detect multiple pathogens and resistance genes within 1 h. We found that introducing this test during limited hours did not improve patient outcomes; rather, achieving optimal clinical impact may depend on continuous availability and effective collaboration with clinical decision-makers. This study emphasizes the need for unlimited access to rapid testing and support from stewardship programs to optimize clinical outcomes from this diagnostic technology in managing bloodstream infections.

## INTRODUCTION

Bacteremia, or bloodstream infection, is a life-threatening condition (1). Rapidly identifying causal pathogens and administering antimicrobials are critical for effective treatment (2). Conventional culture and antimicrobial susceptibility tests take a significant amount of time to produce final results. Thus, some countries have recently approved molecular methods, such as multiplex PCR, for clinical application (3). For example, the FilmArray blood culture identification 2 (BCID2) panel can detect 33 pathogens and 10 antibiotic resistance genes within 1 h (4).

Several studies have evaluated the clinical impact of multiplex PCR tests for the rapid identification of pathogens in patients with bacteremia (5). The antimicrobial stewardship program (ASP) plays a crucial role in facilitating rapid molecular testing to enhance clinical outcomes (6). The Infectious Diseases Society of America recommends using rapid molecular testing in the presence of an ASP (7). However, data on the clinical impacts of rapid multiplex PCR testing for bacteremia remain inconsistent (8–11). In addition to ASP, evidence regarding factors associated with the differential clinical impact of rapid multiplex PCR testing is relatively scarce. In Japan, the FilmArray BCID2 was approved for clinical use in 2020. While the clinical impact of BCID2 among patients with bacteremia has not been reported, a Japanese study reported improved overall clinical outcomes in patients tested with BCID2 (*n* = 139) compared with controls (*n* = 156) (12).

While multiple studies have evaluated the effect of rapid multiplex PCR testing, none have shown a reduction in mortality by implementing BCID2 (13–16). This study aimed to evaluate the clinical impact of rapid multiplex PCR testing among patients with bacteremia, focusing on the stratification by the availability of a rapid BCID2 implementation.

## RESULTS

### Patient characteristics

During the study period, a total of 2,872 hospitalized patients with positive blood cultures were included in the analysis (1,833 in the pre-BCID2 group and 1,039 in the BCID2 group), of which 1,971 had true bacteremia (1,280 in the pre-BCID2 group and 691 in the BCID2 group). For patients with positive blood cultures during weekday daytime hours, there were 920 patients (573 pre-BCID2 and 347 BCID2), of which 609 had true bacteremia (393 pre-BCID2 and 216 BCID2). Participant characteristics are presented in Table 1 (Supplemental Tables S1–S3). The median ages were similar across all groups, ranging from 76.0 to 79.0 years. The most prevalent comorbidities were gastrointestinal/hepatic conditions (22.6%–35.6% across groups) and hematological/oncological conditions (18.7%–23.9% across groups). The proportion of patients requiring intensive care unit (ICU)/high-care unit (HCU) care was similar between pre-BCID2 and BCID2 groups within each stratified analysis.

**TABLE 1:** Characteristics of the study participants (ALL)^*a*^

	Pre-BCID2 group	BCID2 group	*P* value
N	1,833	1,039	
Age	77.0 [66.0–84.0]	77.0 [66.0–84.0]	0.336
Sex (female)	771 (42.1%)	410 (39.5%)	0.180
Chronic comorbidity			
Cardiovascular	80 (4.4%)	63 (6.1%)	0.049
Gastrointestinal/hepatic	415 (22.6%)	236 (22.7%)	0.963
Endocrine/metabolic/nutritional	169 (9.2%)	80 (7.7%)	0.168
Allergy/autoimmune	19 (1.0%)	19 (1.8%)	0.089
Neurological	190 (11.4%)	88 (8.5%)	0.101
Genetic/congenital	5 (0.3%)	1 (0.1%)	0.428
Renal/urogenital	179 (9.8%)	90 (8.7%)	0.351
Immunodeficiency	13 (0.7%)	11 (1.1%)	0.394
Hematological/oncological	406 (22.1%)	200 (19.2%)	0.071
Admission			
Ward	1,406 (76.7%)	754 (72.6%)	0.015
ICU/HCU	427 (23.3%)	285 (27.4%)	0.015
Time from sample collection to pathogen identification	34.1 [22.8–61.0]	31.8 [20.5–58.5]	<0.001
Total antimicrobial days of therapy	9.7 [4.0–18.7]	10.0 [4.0–19.0]	0.493
Days of therapy of carbapenems	0.0 [0.0–3.3]	0.0 [0.0–3.0]	0.973
Days of therapy of anti-MRSA antimicrobials	0.0 [0.0–0.5]	0.0 [0.0–0.0]	0.387
Days of therapy of anti-fungal	0.0 [0.0–0.0]	0.0 [0.0–0.0]	<0.001
Days of antimicrobial spectrum coverage score	74.7 [30.0–143.0]	73.7 [28.0–141.8]	0.976
Length of hospital stay	19.0 [8.0–43.0]	19.0 [8.0–42.0]	0.796
In-hospital mortality	244 (13.3%)	128 (12.3%)	0.488

^*a*^ BCID2, blood culture identification; HCU, high-care unit; ICU, intensive care unit; MRSA, methicillin-resistant *Staphylococcus aureus*.

### BCID2 test results

In the BCID2 group, 316 PCR tests were performed (Supplemental Table S4). The most frequently detected pathogen was *Escherichia coli* (*n* = 86, 27.2%), followed by the *Klebsiella pneumoniae* group (*n* = 37, 11.7%), *Staphylococcus epidermidis* (*n* = 25, 7.9%), and *Staphylococcus aureus* (*n* = 21, 6.6%). Among resistance markers, mecA/C was detected in 20 cases (6.3%), CTX-M in 22 cases (7.0%), vanA/B in 2 cases (0.6%), and IMP in 1 case (0.3%).

### Clinical outcomes

Multivariable regression analyses across the four different populations revealed varying impacts of BCID2 implementation (Tables 2–5). For all patients with positive blood cultures (Table 2), BCID2 implementation was not significantly associated with most clinical outcomes, including length of hospital stay, in-hospital mortality, total antimicrobial days of therapy (DOT), days of antimicrobial spectrum coverage (DASC) score, carbapenem DOT, or anti-methicillin-resistant *S. aureus* (MRSA) DOT. For patients with bacteremia (Table 3), the implementation of BCID2 was associated with a significant increase in total DOT (coefficient: 0.11, 95% CI: 0.02–0.21, *P* = 0.021). However, no significant differences were observed in other outcomes, including mortality. For patients with positive blood cultures during weekday daytime hours (Table 4), the implementation of BCID2 was associated with a significantly reduced in-hospital mortality (adjusted odds ratio [aOR] 0.58, 95% CI: 0.37–0.92, *P* = 0.021). No significant differences were observed in other outcomes. For patients with bacteremia during weekday daytime hours (Table 5), the implementation of BCID2 was associated with a significant reduction in in-hospital mortality (aOR: 0.50, 95% CI: 0.28–0.89, *P* = 0.018). For patients with a positive result during weekday daytime hours, the aOR by the BCID2 implementation was 0.53 (0.10–2.90, *P* = 0.468) for *S. aureus* and 0.39 (0.16–0.97) for *Enterobacteriaceae* (Supplemental Tables S5 and S6).

**TABLE 2:** Multivariable regression analyses (ALL)^*a*^

	Length of hospital stay		In-hospital mortality		Total DOT		DASC score		DOT carbapenems		DOT anti-MRSA	
	Coefficient	*P* value	Coefficient	*P* value	Coefficient	*P* value	Coefficient	*P* value	Coefficient	*P* value	Coefficient	*P* value
BCID2	−0.01 [−0.11, 0.09]	0.859	0.89 [0.71, 1.12]	0.324	0.09 [0.00, 0.17]	0.051	0.05 [−0.06, 0.15]	0.380	−0.02 [−0.20, 0.16]	0.824	0.02 [−0.29, 0.32]	0.901
Age <18 years	−0.35 [−0.62, −0.08]	0.011	0.09 [0.01, 0.64]	0.017	−0.27 [−0.50, −0.03]	0.026	−0.44 [−0.71, −0.16]	0.002	−0.80 [−1.31, −0.28]	0.003	0.25 [−0.56, 1.06]	0.546
Age 60–79 years	−0.25 [−0.39, −0.10]	0.001	1.51 [1.06, 2.15]	0.022	−0.09 [−0.21, 0.04]	0.176	−0.05 [−0.20, 0.10]	0.498	−0.04 [−0.31, 0.22]	0.745	−0.32 [−0.75, 0.11]	0.139
Age ≥80 years	−0.50 [−0.65, −0.35]	<0.001	1.20 [0.83, 1.72]	0.336	−0.47 [−0.60, −0.35]	<0.001	−0.41 [−0.56, −0.26]	<0.001	−0.45 [−0.72, −0.18]	0.001	−0.95 [−1.38, −0.51]	<0.001
Female	−0.02 [−0.11, 0.07]	0.674	0.70 [0.55, 0.88]	0.003	−0.07 [−0.16, 0.01]	0.087	−0.09 [−0.19, 0.01]	0.070	−0.12 [−0.30, 0.05]	0.173	0.00 [−0.29, 0.29]	0.995
ICU/HCU	0.46 [0.34, 0.57]	<0.001	1.87 [1.48, 2.36]	<0.001	0.26 [0.17, 0.36]	<0.001	0.24 [0.12, 0.35]	<0.001	0.55 [0.34, 0.75]	<0.001	0.76 [0.42, 1.09]	<0.001

^*a*^ BCID2, blood culture identification; DASC, days of antimicrobial spectrum coverage; DOT, days of therapy; HCU, high-care unit; ICU, intensive care unit; MRSA, methicillin-resistant *Staphylococcus aureus*.

**TABLE 3:** Multivariable regression analyses (bacteremia)^*a*^

	Length of hospital stay		In-hospital mortality		Total DOT		DASC score		DOT carbapenems		DOT anti-MRSA	
	Coefficient	*P* value	Coefficient	*P* value	Coefficient	*P* value	Coefficient	*P* value	Coefficient	*P* value	Coefficient	*P* value
BCID2	0.02 [−0.10, 0.14]	0.721	0.79 [0.60, 1.06]	0.112	0.11 [0.02, 0.21]	0.021	0.07 [−0.04, 0.18]	0.243	0.04 [−0.16, 0.24]	0.701	0.00 [−0.36, 0.35]	0.985
Age <18 years	−0.05 [−0.41, 0.30]	0.771	0.16 [0.02, 1.18]	0.072	−0.03 [−0.32, 0.25]	0.832	−0.20 [−0.54, 0.14]	0.244	−0.51 [−1.13, 0.12]	0.113	0.53 [−0.52, 1.57]	0.322
Age 60–79 years	−0.39 [−0.57, −0.22]	<0.001	1.38 [0.90, 2.10]	0.140	−0.14 [−0.28, 0.00]	0.053	−0.09 [−0.26, 0.07]	0.264	−0.08 [−0.37, 0.22]	0.601	−0.43 [−0.94, 0.07]	0.092
Age ≥80 years	−0.59 [−0.77, −0.42]	<0.001	1.14 [0.74, 1.77]	0.546	−0.48 [−0.62, −0.34]	<0.001	−0.41 [−0.58, −0.25]	<0.001	−0.49 [−0.79, −0.19]	0.001	−0.95 [−1.47, −0.43]	<0.001
Female	−0.08 [−0.20, 0.03]	0.142	0.73 [0.55, 0.96]	0.024	−0.08 [−0.18, 0.01]	0.076	−0.11 [−0.22, 0.00]	0.047	−0.14 [−0.33, 0.06]	0.180	−0.07 [−0.41, 0.26]	0.667
ICU/HCU	0.46 [0.32, 0.59]	<0.001	1.70 [1.26, 2.27]	<0.001	0.31 [0.20, 0.42]	<0.001	0.26 [0.13, 0.39]	<0.001	0.56 [0.32, 0.79]	<0.001	0.78 [0.38, 1.19]	<0.001

^*a*^ BCID2, blood culture identification; DASC, days of antimicrobial spectrum coverage; DOT, days of therapy; HCU, high-care unit; ICU, intensive care unit; MRSA, methicillin-resistant *Staphylococcus aureus*.

**TABLE 4:** Multivariable regression analyses (weekday daytime shift)^*a*^

	Length of hospital stay		In-hospital mortality		Total DOT		DASC score		DOT carbapenems		DOT anti-MRSA	
	Coefficient	*P* value	Coefficient	*P* value	Coefficient	*P* value	Coefficient	*P* value	Coefficient	*P* value	Coefficient	*P* value
BCID2	−0.14 [−0.31, 0.03]	0.095	0.58 [0.37, 0.92]	0.021	0.09 [−0.06, 0.23]	0.245	0.06 [−0.11, 0.23]	0.513	0.13 [−0.18, 0.44]	0.424	0.12 [−0.45, 0.69]	0.688
Age <18 years	−0.43 [−0.87, 0.00]	0.050	1.00		−0.42 [−0.80, −0.04]	0.030	−0.67 [−1.11, −0.22]	0.003	−1.35 [−2.20, −0.49]	0.002	0.10 [−1.28, 1.48]	0.887
Age 60–79 years	−0.24 [−0.49, 0.01]	0.056	1.30 [0.72, 2.35]	0.379	−0.15 [−0.37, 0.07]	0.173	−0.11 [−0.37, 0.15]	0.406	−0.13 [−0.59, 0.32]	0.566	−0.28 [−1.07, 0.50]	0.479
Age ≥80 years	−0.43 [−0.68, −0.17]	0.001	0.68 [0.36, 1.28]	0.233	−0.57 [−0.79, −0.35]	<0.001	−0.52 [−0.78, −0.26]	<0.001	−0.69 [−1.16, −0.23]	0.003	−0.95 [−1.76, −0.14]	0.022
Female	0.18 [0.01, 0.34]	0.037	0.80 [0.52, 1.23]	0.304	0.00 [−0.15, 0.15]	0.982	−0.01 [−0.18, 0.16]	0.917	−0.04 [−0.35, 0.26]	0.778	0.25 [−0.28, 0.78]	0.347
ICU/HCU	0.36 [0.17, 0.55]	<0.001	1.73 [1.11, 2.68]	0.016	0.21 [0.04, 0.38]	0.015	0.21 [0.02, 0.41]	0.034	0.30 [−0.05, 0.65]	0.098	0.60 [−0.01, 1.21]	0.054

^*a*^ BCID2, blood culture identification; DASC, days of antimicrobial spectrum coverage; DOT, days of therapy; HCU, high-care unit; ICU, intensive care unit; MRSA, methicillin-resistant *Staphylococcus aureus*.

**TABLE 5:** Multivariable regression analyses (bacteremia during weekday daytime hours)^*a*^

	Length of hospital stay		In-hospital mortality		Total DOT		DASC score		DOT carbapenems		DOT anti-MRSA	
	Coefficient	*P* value	Coefficient	*P* value	Coefficient	*P* value	Coefficient	*P* value	Coefficient	*P* value	Coefficient	*P* value
BCID2	−0.20 [−0.40, 0.01]	0.066	0.50 [0.28, 0.89]	0.018	0.08 [−0.08, 0.24]	0.350	0.04 [−0.14, 0.22]	0.657	0.22 [−0.13, 0.58]	0.215	−0.10 [−0.77, 0.58]	0.782
Age <18 years	−0.09 [−0.70, 0.53]	0.785	1.00		−0.03 [−0.50, 0.44]	0.907	−0.30 [−0.85, 0.24]	0.280	−0.96 [−2.07, 0.16]	0.092	0.55 [−1.31, 2.42]	0.559
Age 60–79 years	−0.46 [−0.78, −0.15]	0.004	1.15 [0.58, 2.30]	0.686	−0.20 [−0.44, 0.04]	0.108	−0.15 [−0.43, 0.13]	0.301	−0.13 [−0.65, 0.40]	0.633	−0.18 [−1.12, 0.77]	0.716
Age ≥80 years	−0.54 [−0.85, −0.22]	0.001	0.55 [0.26, 1.18]	0.124	−0.57 [−0.81, −0.34]	<0.001	−0.54 [−0.81, −0.26]	<0.001	−0.81 [−1.33, −0.29]	0.002	−0.78 [−1.75, 0.20]	0.120
Female	0.07 [−0.13, 0.28]	0.476	0.76 [0.45, 1.28]	0.300	−0.01 [−0.17, 0.14]	0.871	−0.02 [−0.20, 0.16]	0.789	−0.11 [−0.47, 0.24]	0.536	−0.01 [−0.64, 0.61]	0.964
ICU/HCU	0.431 [0.05, 0.56]	0.018	1.60 [0.91, 2.80]	0.101	0.30 [0.11, 0.49]	0.002	0.30 [0.08, 0.52]	0.008	0.42 [−0.01, 0.85]	0.054	0.61 [−0.15, 1.36]	0.115

^*a*^ BCID2, blood culture identification; DASC, days of antimicrobial spectrum coverage; DOT, days of therapy; HCU, high-care unit; ICU, intensive care unit; MRSA, methicillin-resistant *Staphylococcus aureus*.

### Patient factors associated with outcomes

Across all analyses, several patient characteristics were consistently associated with clinical outcomes. Advanced age (≥80 years) was associated with shorter hospital stays, reduced antimicrobial use, and decreased mortality in some analyses. Female sex was generally associated with lower mortality rates. Admission to the ICU/HCU was consistently associated with longer hospital stays, higher mortality, and increased antimicrobial use across all populations.

## DISCUSSION

This study evaluated the clinical impact of BCID2 implementation, considering the availability of a rapid BCID2 test and the presence of true bacteremia. The key finding is that BCID2 implementation was associated with a significant reduction in mortality, specifically among patients with bacteremia during weekday daytime hours, when the test was available and ASP recommendations could be promptly implemented. These results are consistent with previous positive findings, which have shown improved clinical outcomes following the introduction of BCID2 (17–19). The stratified analysis revealed important insights. While BCID2 implementation showed no significant mortality benefit in the overall population or among all bacteremia patients, a significant mortality reduction was observed when focusing on patients with positive blood cultures during weekday daytime hours (OR: 0.58, *P* = 0.021), with the strongest effect seen in patients with true bacteremia during these hours (OR: 0.50, *P* = 0.018). This suggests that the clinical impact of rapid molecular testing is highly dependent on the timing of implementation and the availability of ASP support.

The mortality benefit observed specifically during weekday daytime hours can be attributed to several factors. First, during these hours, both BCID2 testing and ASP recommendations were available, enabling rapid pathogen identification and the immediate optimization of antimicrobial therapy. Second, the presence of experienced clinical staff during daytime hours might have facilitated better implementations of ASP recommendations based on BCID2 results. Third, the ability to make real-time clinical decisions during regular working hours likely contributed to improved patient outcomes.

The lack of a significant mortality benefit in the overall population aligns with previous studies that have shown mixed results for the implementation of rapid molecular testing (8–11). This inconsistency may be attributed to the timing limitations of BCID2 availability, differences in patient populations, and variations in local clinical practices.

The restriction of benefit in mortality to weekday daytime hours meant that patients with bacteremia diagnosed during nights or weekends could not benefit from rapid molecular testing, diluting the overall effect. Multiple studies have emphasized the importance of 24/7 availability for rapid diagnostic testing (20–22). Péan de Ponfilly et al. demonstrated that 24/7 multiplex PCR availability was crucial for optimal clinical outcomes in meningitis management (20). Our restriction to business hours meant that 68% of blood cultures could not benefit from rapid identification, substantially diluting the overall clinical impact.

Interestingly, BCID2 implementation was associated with increased total DOT in some analyses, which may reflect more targeted and prolonged antimicrobial therapy based on specific pathogen identification. The significant increase in total DOT following BCID implementation (coefficient: 0.11, 95% CI: 0.02–0.21, *P* = 0.021) in patients with bacteremia warrants careful interpretation. This finding may reflect several underlying mechanisms rather than inappropriate antimicrobial use: first, rapid pathogen identification through BCID might have enabled more targeted, pathogen-specific therapy, potentially leading to longer courses of antimicrobials rather than premature discontinuation of empirical broad-spectrum therapy. In the pre-BCID era, uncertainty regarding the causative organism might have led to earlier discontinuation of antimicrobials in some cases.

Second, BCID results might have provided clinicians with greater confidence in continuing antimicrobial therapy for the appropriate duration, particularly in cases where the specific pathogen identification supported the need for extended treatment. This may represent improved adherence to evidence-based treatment durations rather than excessive use.

Third, the integration of BCID results with ASP recommendations might have led to more individualized treatment approaches, where therapy duration was optimized based on specific pathogen characteristics and resistance profiles, rather than following a one-size-fits-all approach. This suggests that rapid molecular testing leads to more individualized treatment approaches rather than broad-spectrum empirical therapy. Finally, patients who survived, thanks to a rapid BCID2 implementation, might have had a longer course of antimicrobial therapy given their clinical severity, which did not necessarily reflect unnecessary antimicrobial use. However, we acknowledge that increased DOT could also represent a potential negative outcome if it reflects unnecessary prolongation of therapy. Due to the lack of detailed data on specific antimicrobial regimen changes and de-escalation practices in this study, we were limited in determining whether this increase represents appropriate or inappropriate antimicrobial use. Future studies should include a detailed analysis of antimicrobial appropriateness and adherence to guideline-recommended treatment durations to better characterize this relationship.

The clinical impact of rapid diagnostic testing may also be influenced by the distribution of pathogens and resistance patterns in the study population. Previous studies showing the positive clinical impact of BCID2 implementation often involved populations with a higher prevalence of resistant organisms or unusual pathogens, where rapid identification could significantly alter management (23, 24). In our center, the predominance of *E. coli* (27.2%) and *K. pneumoniae* group (11.7%) among BCID2-positive cases, along with a relatively low prevalence of highly resistant organisms (*vanA*/*B*: 0.6%, IMP: 0.3%), might have limited the extent to which rapid testing altered clinical management compared with conventional methods. At our institution, empiric therapy for suspected bacteremia typically follows evidence-based guidelines, with initial broad-spectrum coverage tailored to the patient’s risk factors and clinical presentation. For patients with suspected urinary tract infection-related bacteremia, third-generation cephalosporins (ceftriaxone or cefotaxime) are commonly used as first-line empiric therapy. For patients with healthcare-associated risk factors, severe illness, or suspected multidrug-resistant organisms, carbapenems (meropenem or imipenem) are preferred for initial empiric coverage. In cases of suspected MRSA infection, vancomycin or linezolid is added to the regimen.

De-escalation practices were already well-established prior to BCID implementation, with a systematic review of antimicrobial therapy typically occurring within 48–72 h of culture positivity, based on culture and susceptibility results. This established framework of appropriate empirical coverage, followed by timely de-escalation, might have created a “ceiling effect” where the additional benefit of rapid identification was limited.

Our findings have important implications for healthcare systems considering the implementation of rapid molecular testing for bacteremia. The results suggest that to maximize clinical benefit, rapid molecular testing should be continuously available, not just during regular working hours. The importance of performing rapid molecular tests 24 h a day, 7 days a week, has been reported (13, 20–22). However, implementing the BCID2 panel and appropriately interpreting the results continuously are usually labor-intensive, often requiring a dedicated laboratory technician and an antimicrobial stewardship clinician. Future studies should investigate strategies for implementing rapid molecular tests and accurately and continuously interpreting their results, particularly in resource-limited settings. Additionally, an active ASP that can provide real-time recommendations based on rapid test results is crucial for achieving mortality benefits.

The impact of rapid diagnostic testing may be more pronounced in specific high-risk populations, where timely and targeted antimicrobial therapy is crucial. For instance, patients with neutropenia, specific immunocompromised conditions, severe sepsis, or septic shock might benefit more from rapid diagnostic technologies (25, 26). Future studies should consider stratifying patients based on risk factors and disease severity to identify the subgroups in which BCID2 implementation may have a more significant impact.

We observed an increase in the incidence of both positive blood culture and bacteremia during the BCID2 period compared with the pre-BCID2 period. In Japan, a significant reduction in various infectious diseases, including invasive infections, was reported during the early phase of the COVID-19 pandemic (27, 28), which fell within the pre-BCID2 period in our study. The reduction in transmissible infections between 2020 and 2022 could explain the difference in incidence between the pre-BCID2 and BCID2 periods.

### Limitations

Our study had several limitations. First, we were unable to measure the exact time to optimal antimicrobial treatment due to limitations in our electronic medical records. Previous studies evaluating the clinical impact of the BCID2 panel have measured the time required for optimal antimicrobial therapy (27, 28). Measuring the time to optimal antimicrobial therapy could have provided more granularity of the clinical impacts of the BCID2 panel. This prevented us from assessing one of the key proposed mechanisms by which rapid molecular testing improves outcomes. Second, for the BCID2 panel to have a clinical impact, the recommendations made by the stewardship team based on the test results must be accepted by the attending physician (29). However, since this study did not measure the acceptance rate of the stewardship team’s recommendations, data regarding the implementation status of the BCID2-based recommendations remain lacking. Third, we did not collect detailed data on antimicrobial de-escalation practices or specific changes in antimicrobial regimens based on BCID2 results. Fourth, the single-center design may limit generalizability to other healthcare settings with different pathogen distributions or ASP capabilities.

### Conclusion

Our study demonstrates that BCID2 implementation can significantly reduce mortality in patients with bacteremia; however, this benefit is primarily observed when testing is available during weekday daytime hours with concurrent ASP support. The mortality reduction (OR: 0.50, *P* = 0.018) among patients with bacteremia during weekday daytime hours represents a clinically meaningful improvement.

These findings emphasize the importance of ensuring the continuous availability of rapid molecular testing and ASP support to maximize the clinical benefits of these technologies. Healthcare systems implementing rapid molecular testing for bacteremia should consider round-the-clock availability and robust ASP infrastructure to achieve optimal patient outcomes.

## MATERIALS AND METHODS

### Study design and overview

This retrospective quasi-experimental study was conducted at a single center, Nara Prefecture General Medical Center, Japan, between May 2018 and December 2024. The study participants were patients admitted to the center with at least one set of positive blood cultures during the study period. No age restrictions were applied. For patients with multiple positive blood cultures within 30 days, only the first positive blood culture was included in the study. The Institutional Review Board of Nara Prefecture General Medical Center approved the study protocol and waived the requirement for written informed consent (approval no. 901).

### Microbiological testing

At the center, the BCID2 panel was first introduced in December 2022. During the first month, the panels were implemented retrospectively without influencing clinical management. Regular clinical use of the BCID2 panel began in January 2023. During the BCID2 period, the BCID2 panel was implemented for all Gram-negative bacteremia cases. For other bacteremia cases, a board-certified infectious disease physician decided whether to order the BCID2 panel. The BCID2 was implemented only during weekday daytime hours (8:30–17:15). The pre-BCID2 and BCID2 periods were defined as May 2018–December 2022 and January 2023–December 2024, respectively. In both periods, blood culture bottles (SA culture bottles for aerobic and anaerobic cultures, and PF Plus culture bottles for pediatric specimens; bioMérieux, France) were incubated using the BACT/ALERT 3D System (bioMérieux, France), which operates continuously (24 h a day, 7 days a week). When positive signals are detected, clinicians are immediately notified, and subculturing is initiated by available laboratory staff, including specialized microbiology and general laboratory technicians. However, subsequent microbiological procedures have significant workflow limitations. Gram staining, organism identification using VITEK MS (MALDI-TOF mass spectrometry; bioMérieux, France), and antimicrobial susceptibility testing using VITEK 2 Blue (bioMérieux, France) were performed exclusively during weekday daytime hours (8:30–17:15) by specialized microbiology technicians.

Antimicrobial susceptibility results were obtained using minimal inhibitory concentrations based on breakpoints established by the Clinical Laboratory Standards Institute (30). Blood agar plates (Becton Dickinson, Tokyo, Japan) were used for subculturing.

This workflow means that while positive blood culture signals are detected and immediately reported to clinicians (24/7), definitive organism identification and antimicrobial susceptibility results are only available during weekday daytime hours. For positive blood cultures detected during weekends or after hours, clinicians must wait until the following weekday morning to receive rapid microbial identification results. During the BCID2 period, this rapid molecular testing was also restricted to weekday daytime hours, resulting in a situation where rapid diagnostic tests (BCID2 results, conventional identification, and ASP recommendations) were only available during regular working hours.

### Antimicrobial stewardship

An active ASP was maintained at the center throughout the study period. The ASP team, comprising a board-certified infectious disease physician, a pharmacist, an infection control nurse, and a laboratory technician, reviewed all positive blood culture cases on weekdays and provided recommendations for clinical management, including antimicrobial treatments, to clinicians. These reviews and recommendations were only available during weekday daytime hours due to staffing limitations. In the BCID2 period, ASP recommendations were incorporated with BCID2 panel results to guide clinical decisions. For positive blood cultures detected during weekends or after hours, ASP review and recommendations were delayed until the next weekday, similar to the conventional identification workflow.

### Outcomes and covariates

The outcomes assessed in this study included length of stay, mortality rate during hospitalization, DOT, and DASC score. The DASC score is calculated by combining the DOT with the spectrum score of each antimicrobial (an antimicrobial with a broader spectrum has a higher spectrum score) (31). While patients undergoing concomitant antiviral treatment were not excluded, antiviral agents were excluded from antimicrobial utilization analyses (DOT and DASC calculations). Antimicrobial therapy days were presented as the total antimicrobial DOT, carbapenems DOT, and anti-MRSA antimicrobials DOT. The primary exposure variable was the availability of the BCID2 panel (BCID2 vs pre-BCID2). Covariates included age group (<18 years, 18–49 years, 50–69 years, and ≥70 years), sex, and the requirement for high-level care at admission (admission to an ICU or a HCU).

### Statistical analysis

Patient demographics, microbiological data, and clinical data are presented as median and interquartile ranges for continuous variables and numbers and percentages for binary variables. To compare these characteristics between the pre-BCID2 and BCID2 groups, Wilcoxon rank-sum tests were conducted for continuous variables, while chi-squared tests or Fisher’s exact tests were used for binary outcomes. Multivariable regression models were applied to evaluate the impact of covariates on each outcome across four different populations: (i) all patients with positive blood cultures, (ii) patients with bacteremia (excluding contaminants), (iii) patients with positive blood cultures during weekday daytime hours, and (iv) patients with bacteremia during weekday daytime hours. This stratified analysis was performed to account for the fact that BCID2 testing was only available during weekday daytime hours and to distinguish between true bacteremia and potential contamination cases. In addition, *ad hoc* analyses were conducted for patients with bacteremia during weekday daytime hours by limiting detected pathogens to *Staphylococcus aureus* (the most common pathogen in Gram-positive cocci) and *Enterobacteriaceae* (the most common pathogen group in Gram-negative bacilli). Negative binomial models were employed for count outcomes, given the overdispersion observed in the Poisson regression models, while logistic regression models were utilized for binary outcomes. All statistical analyses were performed using Stata 18.0 (College Station, TX, USA).

### Key points

In this study, clinical efficacy was demonstrated only when the FilmArray Blood Culture Identification Panel 2 was used concurrently with an antimicrobial stewardship program. It is essential to create an environment where clinicians readily adopt the recommendations of antimicrobial stewardship programs, alongside the continuous availability of the FilmArray blood culture identification panel.

## Data Availability

Data supporting the findings of this study are available upon request from the corresponding authors. The data are not publicly available to protect information that can compromise the privacy of the study participants.

## SUPPLEMENTAL MATERIAL

Tables S1 to S6 spectrum.01980-25-s0001.docx Table S1: Characteristics of the study participants (Bacteremia). Table S2: Characteristics of the study participants (Weekday daytime shift). Table S3: Characteristics of the study participants (Bacteremia during weekday daytime hours). Table S4: Details of BCID2 results during the pre-BCID2 and BCID2 periods. Table S5: Multivariable regression analyses (Staphylococcus aureus detected during daytime shifts on weekdays). Table S6: Multivariable regression analyses (Enterobacteriaceae detected during daytime shifts on weekdays).

ASM does not own the copyrights to Supplemental Material that may be linked to, or accessed through, an article. The authors have granted ASM a non-exclusive, world-wide license to publish the Supplemental Material files. Please contact the corresponding author directly for reuse.
