# Supplementary material for: Impact of multiplex polymerase chain reaction testing in patients with bacteremia
Source: Microbiol Spectr. 2025 Sep 24;13(11):e01980-25. doi: 10.1128/spectrum.01980-25 (PMC12584631; doi:10.1128/spectrum.01980-25)
Supplement: Tables S1 to S6 — Table S1: Characteristics of the study participants (Bacteremia). Table S2: Characteristics of the study participants (Weekday daytime shift). Table S3: Characteristics of the study participants (Bacteremia during weekday daytime hours). Table S4: Details of BCID2 results during the pre-BCID2 and BCID2 periods. Table S5: Multivariable regression analyses (Staphylococcus aureus detected during daytime shifts on weekdays). Table S6: Multivariable regression analyses (Enterobacteriaceae detected during daytime shifts on weekdays). [file spectrum.01980-25-s0001.docx]

**Supplementary Information**

**Impact of Multiplex Polymerase Chain Reaction Testing in Patients with Bacteremia**

Daisuke Kitagawa^a,b,#^, Taito Kitano^c^, Takehito Kasamatsu^d^, Naoyuki Shiraishi^d^, Mai Yasuda^e^, Mai Okada^e^, Soma Suzuki^a^, Madoka Sekine^a^, Ryo Yamanishi^a^, Ayu Mukai^a^, Ritsuki Uejima^a^, Yuki Suzuki^b^, Akiyo Nakano^b^, Ryuichi Nakano^b^, Hisakazu Yano^b^, Fumihiko Nakamura^a^, Koichi Maeda^d^

^a^Department of Laboratory Medicine, Nara Prefecture General Medical Center, Japan

^b^Department of Microbiology and Infectious Diseases, Nara Medical University, Japan

^c^Department of Pediatrics, Nara Prefecture General Medical Center, Japan

^d^Department of Infectious Diseases, Nara Prefecture General Medical Center, Japan

^e^Department of Pharmacy, Nara Prefecture General Medical Center, Japan

**#Corresponding authors:**

Daisuke Kitagawa

E-mail: [d.kitagawa.med@gmail.com](about:blank)

Supplemental Table S1. Characteristics of the study participants (Bacterimia)

|  | Pre-BCID2 group | BCID2 group | p value |
| --- | --- | --- | --- |
| N | 1,280 | 691 |  |
| Age | 77.0 [67.0–84.0] | 78.0 [66.4–84.0] | 0.072 |
| Sex (female) | 547 (42.7%) | 268 (38.8%) | 0.094 |
| Chronic comorbidity | | |  |
| Cardiovascular | 46 (3.6%) | 35 (5.1%) | 0.123 |
| Gastrointestinal/Hepatic | 349 (27.3%) | 199 (28.8%) | 0.494 |
| Endocrine/Metabolic/Nutritional | 105 (8.2%) | 57 (8.2%) | 1.000 |
| Allergy/Autoimmune | 14 (1.1%) | 12 (1.7%) | 0.300 |
| Neurological | 106 (8.3%) | 54 (7.8%) | 0.796 |
| Genetic/Congenital | 2 (0.2%) | 0 (0.0%) | 0.544 |
| Renal/Urogenital | 146 (11.4%) | 73 (10.6%) | 0.600 |
| Immunodeficiency | 11 (0.9%) | 8 (1.2%) | 0.630 |
| Hematological/Oncological | 306 (23.9%) | 151 (21.9%) | 0.314 |
| Admission | |  |  |
| Ward | 1020 (79.7%) | 517 (74.8%) | 0.014 |
| ICU/HCU | 260 (20.3%) | 174 (25.2%) | 0.014 |
| Time from sample collection to pathogen identification | 28.3 [21.5−48.4] | 25.1 [17.8−46.1] | < 0.001 |
| Total antimicrobial days of therapy | 11.0 [5.0–19.5] | 11.7 [5.5–20.8] | 0.087 |
| Days of therapy of carbapenems | 0.0 [0.0–3.7] | 0.0 [0.0–3.3] | 0.788 |
| Days of therapy of anti-MRSA antimicrobials | 0.0 [0.0–0.5] | 0.0 [0.0–0.3] | 0.695 |
| Days of therapy of anti-fungal | 0.0 [0.0–0.0] | 0.0 [0.0–0.0] | < 0.001 |
| Days of antimicrobial spectrum coverage score | 84.0 [36.0–148.0] | 85.3 [39.5–148.0] | 0.374 |
| Length of hospital stay | 18.0 [8.0–41.0] | 19.0 [8.0–41.0] | 0.715 |
| In-hospital mortality | 176 (13.8%) | 80 (11.6%) | 0.182 |

Abbreviations: BCID2, blood culture identification; HCU, high-care unit; ICU, intensive care unit; MRSA, methicillin-resistant *Staphylococcus aureus*.

Supplemental Table S2. Characteristics of the study participants (Weekday daytime shift)

|  | Pre-BCID2 group | BCID2 group | p value |
| --- | --- | --- | --- |
| N | 573 | 347 |  |
| Age | 76.0 [64.0–84.0] | 77.0 [66.0–85.0] | 0.112 |
| Sex (female) | 227 (39.6%) | 126 (36.3%) | 0.328 |
| Chronic comorbidity | | |  |
| Cardiovascular | 24 (4.2%) | 18 (5.2%) | 0.516 |
| Gastrointestinal/Hepatic | 121 (21.1%) | 98 (28.2%) | 0.017 |
| Endocrine/Metabolic/Nutritional | 52 (9.1%) | 23 (6.6%) | 0.215 |
| Allergy/Autoimmune | 5 (0.9%) | 6 (1.7%) | 0.348 |
| Neurological | 63 (10.1%) | 27 (7.8%) | 0.136 |
| Genetic/Congenital | 2 (0.3%) | 0 (0.0%) | 0.530 |
| Renal/Urogenital | 63 (10.1%) | 28 (8.1%) | 0.172 |
| Immunodeficiency | 4 (0.7%) | 5 (1.4%) | 0.310 |
| Hematological/Oncological | 129 (22.5%) | 65 (18.7%) | 0.183 |
| Admission | |  |  |
| Ward | 439 (76.6%) | 256 (73.8%) | 0.343 |
| ICU/HCU | 134 (23.4%) | 91 (26.2%) | 0.343 |
| Time from sample collection to pathogen identification | 27.9 [21.8−40.5] | 24.4 [17.5−37.8] | < 0.001 |
| Total antimicrobial days of therapy | 10.0 [5.0–18.8] | 9.0 [4.0–18.8] | 0.364 |
| Days of therapy of carbapenems | 0.0 [0.0–3.7] | 0.0 [0.0–3.3] | 0.896 |
| Days of therapy of anti-MRSA antimicrobials | 0.0 [0.0–1.0] | 0.0 [0.0–0.0] | 0.038 |
| Days of therapy of anti-fungal | 0.0 [0.0–0.0] | 0.0 [0.0–0.0] | 0.031 |
| Days of antimicrobial spectrum coverage score | 74.0 [35.0–144.3] | 68.0 [28.0–143.0] | 0.274 |
| Length of hospital stay | 20.0 [9.0–43.0] | 16.0 [8.0–37.0] | 0.068 |
| In-hospital mortality | 78 (13.6%) | 29 (8.4%) | 0.019 |

Abbreviations: BCID2, blood culture identification; HCU, high-care unit; ICU, intensive care unit; MRSA, methicillin-resistant *Staphylococcus aureus*.

Supplemental Table S3. Characteristics of the study participants (Bacteremia during weekday daytime hours)

|  | Pre-BCID2 group | BCID2 group | p value |
| --- | --- | --- | --- |
| N | 393 | 216 |  |
| Age | 77.0 [67.0–84.0] | 79.0 [68.8–86.0] | 0.032 |
| Sex (female) | 161 (41.0%) | 77 (35.6%) | 0.224 |
| Chronic comorbidity | | |  |
| Cardiovascular | 14 (3.6%) | 9 (4.2%) | 0.825 |
| Gastrointestinal/Hepatic | 101 (25.7%) | 77 (35.6%) | 0.012 |
| Endocrine/Metabolic/Nutritional | 32 (8.1%) | 11 (5.1%) | 0.187 |
| Allergy/Autoimmune | 4 (1.0%) | 2 (0.9%) | 1.000 |
| Neurological | 36 (9.2%) | 13 (6.0%) | 0.213 |
| Genetic/Congenital | 1 (0.3%) | 0 (0.0%) | 1.000 |
| Renal/Urogenital | 49 (12.5%) | 19 (8.8%) | 0.181 |
| Immunodeficiency | 3 (0.8%) | 2 (0.9%) | 1.000 |
| Hematological/Oncological | 93 (23.7%) | 43 (19.9%) | 0.312 |
| Admission | |  |  |
| Ward | 314 (79.9%) | 171 (79.2%) | 0.834 |
| ICU/HCU | 79 (20.1%) | 45 (20.8%) | 0.834 |
| Time from sample collection to pathogen identification | 24.7 [20.3−33.9] | 19.8 [14.8−29.0] | < 0.001 |
| Total antimicrobial days of therapy | 11.5 [6.0–19.8] | 10.7 [5.0–19.8] | 0.730 |
| Days of therapy of carbapenems | 0.0 [0.0–4.0] | 0.2 [0.0–4.0] | 0.675 |
| Days of therapy of anti-MRSA antimicrobials | 0.0 [0.0–1.5] | 0.0 [0.0–0.0] | 0.085 |
| Days of therapy of anti-fungal | 0.0 [0.0–0.0] | 0.0 [0.0–0.0] | < 0.001 |
| Days of antimicrobial spectrum coverage score | 90.0 [45.0–152.5] | 80.2 [37.9–148.0] | 0.429 |
| Length of hospital stay | 20.0 [9.0–43.0] | 16.0 [8.0–36.5] | 0.145 |
| In-hospital mortality | 58 (14.8%) | 17 (7.9%) | 0.014 |

Abbreviations: BCID2, blood culture identification; HCU, high-care unit; ICU, intensive care unit; MRSA, methicillin-resistant *Staphylococcus aureus*.

| Supplemental Table S4. Details of BCID2 results during the pre-BCID2 and BCID2 periods | | | |
| --- | --- | --- | --- |
| Categories | Pre-BCID2 group | BCID2 group |  |
| Number of PCR tests performed | | N/A | 316 |
| Gram-Positive Bacteria | |  |  |
| *Enterococcus faecalis* | | NA | 12 (3.8%) |
| *Enterococcus faecium* | | NA | 14 (4.4%) |
| *Listeria monocytogenes* | | NA | 0 |
| *Staphylococcus aureus* | | NA | 21 (6.6%) |
| *Staphylococcus epidermidis* | | NA | 25 (7.9%) |
| *Staphylococcus lugdunensis* | | NA | 3 (0.9%) |
| Other *Staphylococcus* spp. | | NA | 20 (6.3%) |
| *Streptococcus agalactiae* | | NA | 8 (2.5%) |
| *Streptococcus pneumoniae* | | NA | 6 (1.9%) |
| *Streptococcus pyogenes* | | NA | 3 (0.9%) |
| Other *Streptococcus* spp. | | NA | 14 (4.4%) |
| Gram-Negative Bacteria | |  |  |
| *Acinetobacter baumannii* complex | | NA | 5 (1.6%) |
| *Bacteroides fragilis* | | NA | 5 (1.6%) |
| *Enterobacter cloacae* complex | | NA | 9 (2.8%) |
| *Escherichia coli* | | NA | 86 (27.2%) |
| *Klebsiella aerogenes* | | NA | 7 (2.2%) |
| *Klebsiella oxytoca* | | NA | 4 (1.3%) |
| *Klebsiella pneumoniae* group | | NA | 37 (11.7%) |
| *Proteus* spp. | | NA | 5 (1.6%) |
| *Salmonella* spp. | | NA | 1 (0.3%) |
| *Serratia marcescens* | | NA | 4 (1.3%) |
| Other *Enterobacterales* | | NA | 1 (0.3%) |
| *Haemophilus influenzae* | | NA | 2 (0.6%) |
| *Neisseria meningitidis* | | NA | 0 |
| *Pseudomonas aerugimosa* | | NA | 15 (4.7%) |
| *Stenotrophomonas maltophilia* | | NA | 4 (1.3%) |
| *Yeast* | |  |  |
| *Candida albicans* | | NA | 10 (3.2%) |
| *Candida auris* | | NA | 0 |
| *Candida glabrata* | | NA | 5 (1.6%) |
| *Candida krusei* | | NA | 0 |
| *Candida parapsilosis* | | NA | 3 (0.9%) |
| *Candida tropicalis* | | NA | 3 (0.9%) |
| *Cryptococcus neoformans/gattii* | | NA | 0 |
| Antimicrobial Resistance Gene | |  |  |
| IMP | | NA | 1 (0.3%) |
| KPC | | NA | 0 |
| OXA-48-like | | NA | 0 |
| NDM | | NA | 0 |
| VIM | | NA | 0 |
| *mcr-1* | | NA | 0 |
| CTX-M | | NA | 22 (7.0%) |
| *mecA/C* | | NA | 20 (6.3%) |
| *mecA/C* and MRE/J | | NA | 14 (4.4%) |
| *vanA/B* | | NA | 2 (0.6%) |

Antimicrobial Resistance Gene Mechanisms: IMP, KPC, OXA-48-like, NDM, VIM: Carbapenemase genes conferring resistance to carbapenem antibiotics. *mcr-1*: Mobilized colistin resistance gene conferring resistance to polymyxin antibiotics. CTX-M: Extended-spectrum β-lactamase gene conferring resistance to extended-spectrum cephalosporins. *mecA/C*: Methicillin resistance genes in staphylococci conferring resistance to β-lactam antibiotics. *mecA/C* and MRE/J: Combined methicillin resistance genes with additional resistance determinants. *vanA/B*: Vancomycin resistance genes in enterococci conferring high-level vancomycin resistance. The presence of these resistance genes suggests potential treatment challenges and guides targeted antimicrobial therapy selection.

Supplemental Table S5. Multivariable regression analyses (*Staphylococcus aureus* detected during daytime shifts on weekdays)

|  | Length of hospital stay | | In-hospital mortality | | Total DOT | | DASC score | | DOT carbapenems | | DOT anti-MRSA | |
| --- | --- | --- | --- | --- | --- | --- | --- | --- | --- | --- | --- | --- |
|  | Coefficient | p | Coefficient | p | Coefficient | p | Coefficient | p | Coefficient | p | Coefficient | p |
| BCID2 | -0.14 [-0.85, 0.56] | 0.693 | 0.53 [0.10, 2.90] | 0.468 | 0.36 [-0.18, 0.90] | 0.190 | 0.17 [-0.48, 0.82] | 0.609 | 0.65 [-1.03, 2.34] | 0.447 | 0.82 [-0.96, 2.60] | 0.365 |
| Age < 18 years | 0.62 [-0.43, 1.67] | 0.248 | 1.00 |  | 0.45 [-0.41, 1.32] | 0.304 | -0.03 [-1.06, 1.01] | 0.958 | -17.79 [-3250.49, 3214.90] | 0.991 | 2.46 [0.01, 4.93] | 0.05 |
| Age 60–79 years | 0.24 [-0.57, 1.05] | 0.564 | 0.92 [0.21, 4.13] | 0.916 | 0.16 [-0.53, 0.84] | 0.654 | 0.02 [-0.80, 0.84] | 0.957 | -0.58 [-2.77, 1.60] | 0.601 | 1.89 [-0.02, 3.81] | 0.053 |
| Age ≥ 80 years | 0.22 [-0.64, 1.09] | 0.615 | 0.34 [0.06, 1.98] | 0.230 | -0.23 [-0.92, 0.46] | 0.516 | -0.32 [-1.14, 0.50] | 0.443 | -1.41 [-3.56, 0.75] | 0.2 | 0.38 [-1.63, 2.40] | 0.708 |
| Female | 0.15 [-0.41, 0.71] | 0.603 | 0.22 [0.04, 1.14] | 0.071 | 0.19 [-0.27, 0.65] | 0.421 | 0.09 [-0.47, 0.65] | 0.749 | -1.07 [-2.86, 0.72] | 0.243 | -0.27 [-1.78, 1.23] | 0.721 |
| ICU/HCU | -0.39 [-1.00, 0.22] | 0.208 | 0.76 [0.19, 3.01] | 0.695 | -0.15 [-0.65, 0.35] | 0.561 | -0.23 [-0.83, 0.36] | 0.443 | -0.29 [-2.13, 1.55] | 0.757 | -1.31 [-2.78, 0.17] | 0.083 |

Abbreviations: BCID2, blood culture identification; DASC, days of antimicrobial spectrum coverage; DOT, days of therapy; HCU, high-care unit; ICU, intensive care unit; MRSA, methicillin-resistant *Staphylococcus aureus*.

Supplemental Table S6. Multivariable regression analyses (Enterobacteriaceae detected during daytime shifts on weekdays)

|  | Length of hospital stay | | In-hospital mortality | | Total DOT | | DASC score | | DOT carbapenems | | DOT anti-MRSA | |
| --- | --- | --- | --- | --- | --- | --- | --- | --- | --- | --- | --- | --- |
|  | Coefficient | p | Coefficient | p | Coefficient | p | Coefficient | p | Coefficient | p | Coefficient | p |
| BCID2 | -0.21 [-0.50, 0.08] | 0.151 | 0.39 [0.16, 0.97] | 0.043 | 0.10 [-0.11, 0.31] | 0.370 | 0.12 [-0.14, 0.37] | 0.365 | 0.16 [-0.28, 0.59] | 0.48 | -0.78 [-2.01, 0.44] | 0.211 |
| Age < 18 years | -0.21 [-1.62, 1.20] | 0.770 | 1.00 |  | 0.55 [-0.49, 1.58] | 0.300 | 0.20 [-1.05, 1.46] | 0.753 | -0.78 [-3.06, 1.50] | 0.504 | 1.36 [-4.17, 6.88] | 0.631 |
| Age 60–79 years | -0.63 [-1.12, -0.14] | 0.012 | 1.77 [0.47, 6.65] | 0.396 | 0.27 [-0.09, 0.63] | 0.139 | 0.24 [-0.19, 0.66] | 0.281 | 0.18 [-0.54, 0.90] | 0.616 | 0.53 [-1.41, 2.47] | 0.592 |
| Age ≥ 80 years | -0.83 [-1.32, -0.34] | 0.001 | 0.79 [0.20, 3.21] | 0.743 | -0.15 [-0.51, 0.21] | 0.427 | -0.24 [-0.67, 0.19] | 0.268 | -0.51 [-1.24, 0.23] | 0.175 | -0.33 [-2.38, 1.71] | 0.749 |
| Female | -0.06 [-0.35, 0.23] | 0.698 | 1.01 [0.46, 2.22] | 0.974 | -0.03 [-0.25, 0.18] | 0.761 | -0.05 [-0.30, 0.20] | 0.683 | -0.08 [-0.51, 0.35] | 0.726 | 0.24 [-0.98, 1.47] | 0.696 |
| ICU/HCU | 0.67 [0.29, 1.04] | <0.001 | 2.65 [1.14, 6.15] | 0.023 | 0.43 [0.15, 0.70] | 0.002 | 0.45 [0.12, 0.77] | 0.007 | 0.51 [-0.04, 1.05] | 0.067 | 1.43 [-0.10, 2.97] | 0.067 |

Abbreviations: BCID2, blood culture identification; DASC, days of antimicrobial spectrum coverage; DOT, days of therapy; HCU, high-care unit; ICU, intensive care unit; MRSA, methicillin-resistant *Staphylococcus aureus*.
